# Integrated Regulatory and Metabolic Networks of the Marine Diatom *Phaeodactylum tricornutum* Predict the Response to Rising CO_2_ Levels

**DOI:** 10.1128/mSystems.00142-16

**Published:** 2017-02-14

**Authors:** Jennifer Levering, Christopher L. Dupont, Andrew E. Allen, Bernhard O. Palsson, Karsten Zengler

**Affiliations:** a Department of Bioengineering, University of California San Diego, La Jolla, California, USA; b Microbial and Environmental Genomics, J. Craig Venter Institute, La Jolla, California, USA; c Integrative Oceanography Division, Scripps Institute of Oceanography, University of California San Diego, La Jolla, California, USA; University of Michigan

**Keywords:** *Phaeodactylum tricornutum*, coregulated genes, genome-scale metabolic network reconstruction, integrated network modeling, regulatory network inference

## Abstract

Diatoms are eukaryotic microalgae that are responsible for up to 40% of the ocean’s primary productivity. How diatoms respond to environmental perturbations such as elevated carbon concentrations in the atmosphere is currently poorly understood. We developed a transcriptional regulatory network based on various transcriptome sequencing expression libraries for different environmental responses to gain insight into the marine diatom’s metabolic and regulatory interactions and provide a comprehensive framework of responses to increasing atmospheric carbon levels. This transcriptional regulatory network was integrated with a recently published genome-scale metabolic model of *Phaeodactylum tricornutum* to explore the connectivity of the regulatory network and shared metabolites. The integrated regulatory and metabolic model revealed highly connected modules within carbon and nitrogen metabolism. *P. tricornutum*’s response to rising carbon levels was analyzed by using the recent genome-scale metabolic model with cross comparison to experimental manipulations of carbon dioxide.

**IMPORTANCE** Using a systems biology approach, we studied the response of the marine diatom *Phaeodactylum tricornutum* to changing atmospheric carbon concentrations on an ocean-wide scale. By integrating an available genome-scale metabolic model and a newly developed transcriptional regulatory network inferred from transcriptome sequencing expression data, we demonstrate that carbon metabolism and nitrogen metabolism are strongly connected and the genes involved are coregulated in this model diatom. These tight regulatory constraints could play a major role during the adaptation of *P. tricornutum* to increasing carbon levels. The transcriptional regulatory network developed can be further used to study the effects of different environmental perturbations on *P. tricornutum*’s metabolism.

## Abstract (precis)

Using a systems biology approach, we studied the response of the marine diatom *Phaeodactylum tricornutum* to changing atmospheric carbon concentrations on an ocean-wide scale. By integrating an available genome-scale metabolic model and a newly developed transcriptional regulatory network inferred from transcriptome sequencing expression data, we demonstrate that carbon metabolism and nitrogen metabolism are strongly connected and the genes involved are coregulated in this model diatom. These tight regulatory constraints could play a major role during the adaptation of *P. tricornutum* to increasing carbon levels. The transcriptional regulatory network developed can be further used to study the effects of different environmental perturbations on *P. tricornutum*’s metabolism.

## INTRODUCTION

The carbon dioxide (CO_2_) concentration in the atmosphere is expected to double by the end of the century because of fossil fuel burning and land use changes resulting in an increase in dissolved carbon levels and acidification of the oceans (1).

Diatoms are photosynthetic eukaryotic microalgae that are ubiquitous in marine and freshwater habitats (2). It is estimated that diatoms are responsible for up to 40% of all photosynthetic carbon fixation in the sea and thus are crucial for the global carbon cycle (3, 4). Understanding these unicellular organisms in detail to predict their response to environmental changes such as rising CO_2_ levels is therefore of high importance when evaluating the future global carbon budget.

Here, we investigated the metabolic response of *Phaeodactylum tricornutum*, a model diatom, to increasing CO_2_ concentrations on an ocean-wide scale. Diatoms satisfy their carbon requirements by utilizing both CO_2_ and bicarbonate (HCO_3_), the former through concentration-forced diffusion into the cytoplasm and the latter through active ion transporters (5). Rising atmospheric CO_2_ levels will have two effects on the surface ocean, a slightly decreased pH and a higher total dissolved inorganic carbon (DIC) level. DIC is a combination of CO_2_, HCO_3_, and carbonate. The relative amounts of the three DIC species are set by the pH. At modern seawater pHs, the most dominant form of DIC is HCO_3_. Under bloom conditions, the pH increases, resulting in a rising carbonate content but also a lower total DIC content. Under ocean acidification conditions, there is a higher total DIC level but also the percentage of CO_2_ increases, essentially resulting in ocean carbonation. Overall, future atmospheric increases can be expected to facilitate higher CO_2_ diffusive uptake through decreased pH and active HCO_3_ uptake through increased total DIC.

So far, it is not understood how diatoms’ metabolism will respond to increasing atmospheric CO_2_ levels. Here, we exploited a genome-scale metabolic network recently developed for the model diatom *P. tricornutum* to understand its metabolic response to rising CO_2_ concentrations (6). Genome-scale metabolic network reconstructions are based on the organism’s annotated genome and comprise information about the metabolic reactions and the gene products by which they are catalyzed. These models allow detailed analysis of the organism’s physiology, facilitate metabolic engineering efforts, and enable predictions of physiological changes in response to environmental perturbations (7, 8).

Within this study, the genome-scale metabolic network for *P. tricornutum* was used to determine which metabolic subsystems are affected by increased CO_2_ levels. For our simulations, we assumed a global ocean where carbon is becoming more bioavailable while nitrate delivery to the surface of the ocean will be relatively invariant. To resemble modern seawater, carbon can be taken up in the form of HCO_3_, the most dominant form of DIC at the current seawater pH. Increased atmospheric CO_2_ concentrations were simulated by mimicking the effects of ocean acidification and increasing the boundaries of HCO_3_ uptake in the model while not allowing nitrate uptake rates to rise concordantly. We combined these results with a differential gene expression analysis and a transcriptional regulatory network (TRN) inferred from transcriptome sequencing (RNA-Seq) data in order to gain a comprehensive understanding of the interconnection between metabolic and regulatory mechanisms that could drive the adaptation to increased CO_2_ concentrations.

## RESULTS AND DISCUSSION

### Integrated regulatory and metabolic model reveals highly connected modules in carbon and nitrogen metabolism.

Our previous efforts include a genome-scale metabolic network reconstruction for *P. tricornutum* that allows detailed insights into its physiology and provides a framework to analyze and predict genotype-phenotype relationships (6). In contrast, TRNs point out connections among the environment, genotype, and expression state and facilitate prediction of the global transcriptional responses to environmental and genetic perturbations (9). To gain more complete insights into the interactions between metabolic and regulatory mechanisms, a TRN for *P. tricornutum* was inferred. For an overview of the interaction between the regulatory network and the genome-scale metabolic network, see Fig. S1 in the supplemental material.

10.1128/mSystems.00142-16.6 FIG S1  Interaction between the regulatory network and the genome-scale metabolic network. As an example, one bicluster of the TRN (left; experimental conditions are omitted) and the reaction catalyzed by one of the genes and the corresponding information in the genome-scale model (right) are shown. The TRN consists of coexpressed genes and experimental conditions organized into biclusters. The metabolic network contains information about the metabolic reactions and the gene products (GPRs) by which they are catalyzed. Both networks interact via the metabolic genes. By using the information included in the genome-scale model, subsystems are associated with the genes in the TRN. Download FIG S1, TIF file, 0.3 MB. Copyright © 2017 Levering et al. 2017 Levering et al. This content is distributed under the terms of the Creative Commons Attribution 4.0 International license.

In a first step, sets of genes that are putatively coregulated in subsets of environmental conditions were identified on the basis of a large set of RNA-Seq data for diverse environmental conditions, such as distinct CO_2_ or iron levels (Table 1); genomic information; and protein-protein interaction data (see Materials and Methods). Subsequently, we inferred regulatory influences of transcription factors (TFs) on the coregulated genes. The resulting TRN can be used to analyze how TFs induce genome-wide transcriptional responses, i.e., activation or repression of transcription, to environmental perturbations. Genes that are coregulated throughout different physiological conditions are likely involved in the same biological processes. In order to identify potential biological processes that are carried out by the coregulated gene sets, a gene ontology (GO) enrichment analysis was performed.

**TABLE 1:** Overview of RNA-Seq libraries used to infer the global regulatory network of *P. tricornutum*

ID	Experimental conditions	No. of samples	Accession no.
GABA/DD	Exponential growth, diatoms treated with two concentrations of either 2-*trans*-4-*trans*-decadienal or γ-aminobutyric acid and sampled over time	44	SAMN05925108–SAMN05925151
CO_2_	Duplicate cultures at 1,000 and 150 ppm CO_2_	4	Table S5
CO_2_ dark/light	Triplicate cultures at 50, 400, and 5,000 ppm CO_2_ under dark and light conditions	18	SAMN05176215–SAMN05176250
N short term	N-starved cells given different N sources and monitored in the very short term	30	SAMN04488978–SAMN04489007
Pulse-chase	Duplicate culture grown on urea or nitrate and harvested in exponential growth phase	4	SAMN05925158–SAMN05925161
N sources	Cultures grown on 880 μM NO_3_, 75 μM NO_3_, 880 μM NH_4_, 75 μM NH_4_, 880 μM urea, and 37.5 μM urea; high-nitrogen cultures harvested during exponential growth; low-nitrogen cultures harvested at onset of stationary phase	6	SAMN05925152–SAMN05925157
B_12_	Cultures grown with or without vitamin B_12_ at a high or low Fe concentration	8	SRX142057, SRX142058, SRX142055, SRX142059, SRX142060, SRX142061, SRX142086, SRX142087
Fe diel	Cultures grown at 15.0, 30.0, or 300.0 nM total Fe and sampled over a diel cycle	49	SAMN04461541–SAMN04461589
GSA/MSX/Rapa	Experiment examining response to glufosinate, sirolimus, or l-methionine-dl-sulfoximine	16	SAMN05925188–SAMN05925203

By using the approach described, 1,214 metabolic genes and TFs were grouped into 121 biclusters with a mean of 20 genes per cluster (see Table S1). For 118 of the clusters, possible regulators could be predicted. On average, these 118 clusters are regulated by 10 TFs, whereas 69 clusters are regulated by more than 10 TFs. The maximal number of TFs predicted to regulate one cluster is 22.

10.1128/mSystems.00142-16.1 TABLE S1  Overview of the inferred TRN. For each bicluster, the genes, experimental conditions, enriched GO terms, predicted motifs, and regulators (with strength of influence [negative, repression; positive, activation]), as well as whether they are enriched in genes involved in carbon and/or nitrogen metabolism, are listed. Download TABLE S1, XLSX file, 0.1 MB. Copyright © 2017 Levering et al. 2017 Levering et al. This content is distributed under the terms of the Creative Commons Attribution 4.0 International license.

10.1128/mSystems.00142-16.2 TABLE S2  Annotations associated with the regulon for each cluster. For each cluster, the genes and the reaction association and annotation (reaction EC number, KEGG ID, subsystem association), as given in the genome-scale model, as well as the Ensembl or NCBI database (for genes located on organelle genomes) annotation, are listed. Download TABLE S2, XLSX file, 0.5 MB. Copyright © 2017 Levering et al. 2017 Levering et al. This content is distributed under the terms of the Creative Commons Attribution 4.0 International license.

10.1128/mSystems.00142-16.3 TABLE S3  Abbreviations used for the experimental conditions in Table S1. Download TABLE S3, XLSX file, 0.02 MB. Copyright © 2017 Levering et al. 2017 Levering et al. This content is distributed under the terms of the Creative Commons Attribution 4.0 International license.

On the basis of the TRN (see Table S1), genes taking part in carbon and nitrogen metabolism are very often regulated by the same TFs in *P. tricornutum*. To investigate this strong correlation between carbon metabolism and nitrogen metabolism more precisely, we performed a GO enrichment analysis and associated each significant GO term with carbon or nitrogen metabolism. By using the subsystems given in the model, genes associated with reactions involved in amino acid metabolism, nitrogen metabolism, nucleotide metabolism, or the urea cycle were categorized into nitrogen metabolism. Genes associated with reactions involved in aldehyde degradation, ascorbate metabolism, butanoate metabolism, the Calvin-Benson cycle, carbon fixation, galactose metabolism, nucleotide sugar metabolism, oxidative phosphorylation, photosynthesis, pentose interconversions, the pentose phosphate pathway, or pyruvate metabolism were categorized as carbon metabolism. Genes associated with reactions involved in the tricarboxylic acid cycle were classified as both carbon metabolism and nitrogen metabolism. On the basis of this mapping, 48 clusters were enriched or purified in genes involved in carbon metabolism and 40 clusters contain enriched or purified genes taking part in nitrogen metabolism. Twenty-two clusters are enriched in genes involved in both carbon metabolism and nitrogen metabolism. Of the 18 clusters containing nitrogen but not carbon metabolism genes, 9 are not enriched in genes belonging to any other subsystem, 1 is also enriched in genes involved in transport, 1 is also enriched in genes belonging to glycan metabolism, 3 are also enriched for genes involved in cofactor metabolism, and 4 also contain enriched or purified genes involved in lipid metabolism.

While a review of all 22 clusters enriched in genes involved in both carbon metabolism and nitrogen metabolism is not in the scope of this publication, several were examined for previously unmentioned transcriptional and regulatory links. Cluster 15 is enriched in genes involved in pyruvate metabolism, including a putative mitochondrial transporter for pyruvate, nitrogen metabolism, and amino acid metabolism. This cluster contains the mitochondrion-localized urease, which is involved in both anabolic nitrogen metabolism and catabolic nitrogen metabolism (10), and a glutamine dehydrogenase. Other components include the beta-oxidation of fatty acids, phosphoenolpyruvate carboxylase (PEPC, a protein associated with a putative biochemical carbon-concentrating mechanism), and lactate dehydrogenase. Almost all of the predicted proteins in this cluster are involved in carbon and nitrogen metabolism of central and simple metabolites but are remarkably localized in multiple compartments. In hypothetical models of the diatom biochemical carbon-concentrating mechanism, PEPC performs the carboxylation reaction either in the mitochondria or in the periplastidic space while PEPC kinase (PEPCK) is required in the mitochondria (11). PEPCK is found in cluster 69, which is also enriched in glycolysis/gluconeogenesis, the pentose phosphate pathway, amino acid metabolism (including a glutamine dehydrogenase separate from that found in cluster 15), and peroxisomal beta-oxidation of lipids. All told, clusters 15 and 69, encoding 17 and 16 predicted proteins, respectively, code for a substantial portion of the reactions in central metabolism and share multiple metabolites, including ammonia, glutamine, glutamate, pyruvate, phosphoenolpyruvate, oxaloacetate, glycolate, and CO_2_. However, they exhibit different expression patterns indicated by their clustering into different modules. Not surprisingly, the two clusters share two predicted regulatory proteins, J49099 and J47726, that provide a tangible link between transcriptional regulation and metabolite exchange between different pathways and subcellular compartments. Metabolite exchange between subcellular compartments has been shown to be important to diatom metabolism (12), though the exact currencies and coordination are not known. Sharing of regulatory proteins by clusters of genes that code for proteins from multiple compartments that participate in central carbon and nitrogen metabolism provides a possible genetic mechanism for subcellular compartment metabolite exchange.

**FIG 1:**
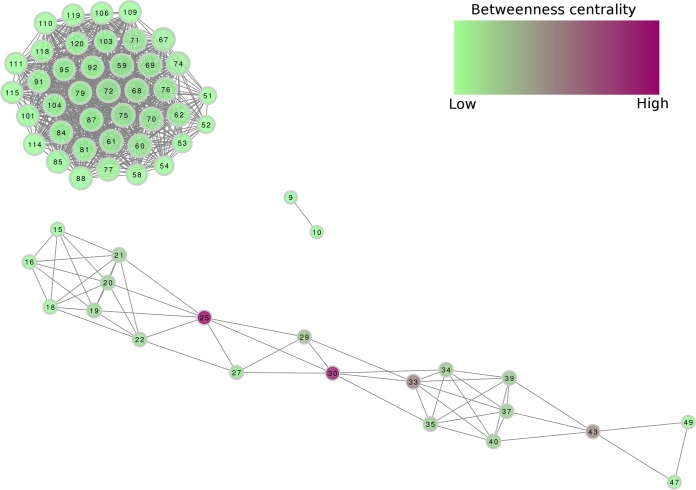
Visualization of the metabolic network connecting transcriptional clusters in *P. tricornutum*. Nodes are transcriptional clusters, while edges show strong connectivity in terms of products in the genome-scale metabolic model (6).

By performing a connectivity analysis based on the clustering result obtained from cMonkey_2_ (13, 14) and the *P. tricornutum* genome-scale model (6) (see Materials and Methods), we could indeed show that many of the clusters share metabolites (see Table S4). We filtered for all carbon- and/or nitrogen-enriched clusters and visualized the connectivity of this subset of 66 clusters with the transcriptional clusters represented as nodes and the shared metabolites represented as edges (Fig. 1). The product network consists of three separate modules, one with only two nodes, one with 20, and one with 44. A nearly identical topology was obtained for the substrates, though the large module was broken into two modules. The subnetwork with 20 nodes includes 4 nodes with moderate to high betweenness centrality, namely, 43, 33, 30, and 25. Cluster 43 contains both photosystems and ribulose bisphosphate carboxylase/oxygenase, highlighting the nonsurprising centrality of photosynthesis to both metabolism and the regulation of gene expression. Cluster 25 contains basic reactions in purine, pyrimidine, and branched-chain amino acid metabolism. Cluster 30 contains chlorophyll biosynthesis and photosynthetic electron transporter, while cluster 33 contains a mixture of TAG biosynthesis, amino acid metabolism, and oxidative phosphorylation. More surprising is the module consisting of just two nodes, 9 and 10. However, these nodes contain a host of reactions that influence not only carbon and nitrogen metabolism but also phosphate metabolism and transport and CO_2_ sensing and uptake. These two nodes also bridge nearly every cellular compartment, including the cytoplasm, mitochondria, peroxisome, and chloroplast. Finally, these two nodes do share a predicted regulator, Phatr3_44139, with a divergent regulation.

10.1128/mSystems.00142-16.4 TABLE S4  Strongly connected clusters. On the basis of the clustering results and the *P. tricornutum* genome-scale model, a cluster-gene reaction product association list was compiled. The connectivity of two clusters was defined as the number of common products divided by the total number of products in both clusters. Clusters with a connectivity of >0.9 are categorized as highly connected. Download TABLE S4, XLSX file, 0.02 MB. Copyright © 2017 Levering et al. 2017 Levering et al. This content is distributed under the terms of the Creative Commons Attribution 4.0 International license.

10.1128/mSystems.00142-16.5 TABLE S5  Raw counts of duplicate cultures at 1,000 and 150 ppm CO_2_. Download TABLE S5, XLSX file, 0.5 MB. Copyright © 2017 Levering et al. 2017 Levering et al. This content is distributed under the terms of the Creative Commons Attribution 4.0 International license.

### Elevated carbon conditions affect *P. tricornutum*’s metabolism.

**FIG 2:**
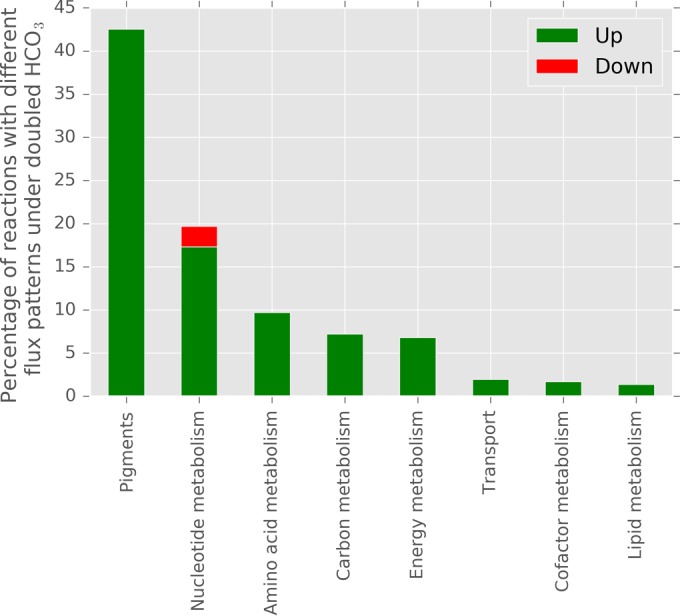
Percentages of reactions showing different fluxes at doubled HCO_3_ levels per group as identified by the solution space sampling of the genome-scale metabolic model of *P. tricornutum*. Pigment metabolism contains most of the reactions with different fluxes under the two conditions, i.e., 43% of the reactions in this group were upregulated at high HCO_3_ concentrations, followed by nucleotide metabolism (19% in total; 17% upregulated and 2% downregulated) and amino acid metabolism (10%). Three reactions, namely, ITCY_c, CMP_c, and ITPA_c, belonging to nucleotide metabolism were downregulated at high HCO_3_ concentrations.

The genome-scale model of *P. tricornutum* (6) was used to evaluate the TRN. In a first step, we investigated the effect of rising atmospheric carbon levels on the diatom’s metabolism. By sampling the solution space of the metabolic model (see Materials and Methods), we identified 175 out of 3,904 reactions showing different flux distributions at low and high HCO_3_ levels. Every flux distribution describes a different state of the genome-scale model representing a different metabolic phenotype (see reference 15 for more details on the simulation and analysis of metabolic networks). The 175 identified reactions belong to 28 out of the 90 subsystems that are accounted for in the metabolic network. Of these 175 reactions, 38 were not gene associated and therefore not taken into account in further analyses. The 137 remaining reactions with different flux patterns at low (1.57 mM) and doubled (3.14 mM) HCO_3_ levels are associated with 141 genes in the metabolic network, and most of their fluxes are elevated at high HCO_3_ concentrations (three reactions, namely, ITCY_c, CMP_c, and ITPA_c, belonging to nucleotide metabolism are downregulated at high HCO_3_ concentrations). Note that we used the biomass objective function (BOF) and the constraints previously determined experimentally (6). The metabolic response to increasing HCO_3_ levels qualitatively predicts *P. tricornutum*’s response to rising atmospheric CO_2_ levels but does not reflect quantitative changes (15). The subsystems were further categorized into groups, e.g., carbon or lipid metabolism. As shown in Fig. 2, the 137 reactions belong to eight different groups (amino acid metabolism, carbon metabolism, cofactor metabolism, energy metabolism, lipid metabolism, nucleotide metabolism, pigments, and transport).

To simulate the effect of rising HCO_3_ levels on *P. tricornutum*’s metabolism, the same constraints on nutrient uptake (except for HCO_3_), product secretion, and biomass composition were used under low- and high-carbon conditions. The biomass function accounts for all known biomass components, i.e., DNA, RNA, protein, pigments, carbohydrates, membrane lipids, and the storage lipids triacylglycerols (TAGs), as well as energetic requirements and their fractional contributions to the overall cellular biomass. As depicted in Fig. 2, these are exactly the subsystems showing reactions with higher fluxes at elevated HCO_3_ levels. Thus, the reaction fluxes are increased to meet the larger demand of biomass components accounting for *P. tricornutum*’s increased growth at increased HCO_3_ levels.

**FIG 3:**
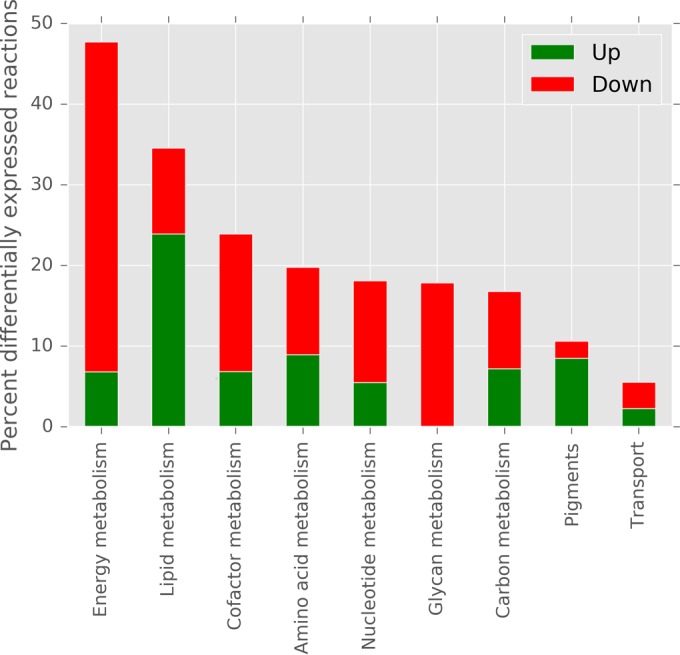
Percentages of reactions differentially expressed at high versus low CO_2_ concentrations per group. Energy metabolism contains most of the differentially expressed reactions; i.e., 40.9% of the reactions in this group were upregulated and 6.8% were downregulated at high CO_2_ concentrations, followed by lipid and cofactor metabolism.

Differential expression analysis was performed to validate and complement the genome-scale model solution space sampling results. In total, 187 differentially expressed genes were identified by comparing gene expression at high and normal CO_2_ concentrations. Half of the genes (93 genes) were downregulated at high CO_2_ concentrations, whereas the other half (94 genes) were upregulated. By using the *P. tricornutum* genome-scale model, the genes were mapped to 1,323 metabolic reactions belonging to 63 model subsystems, which were further categorized into nine groups (amino acid metabolism, carbon metabolism, cofactor metabolism, energy metabolism, glycan metabolism, lipid metabolism, nucleotide metabolism, pigments, and transport) (Fig. 3). Note that the genome-scale model includes a very detailed lipid metabolism in which each elongation and degradation step is modeled separately, although many of these steps are catalyzed by the same gene. This fact explains the high number of reactions mapped to the 187 differentially expressed genes. These 187 genes include 35 genes mapped to 1,147 reactions involved in lipid metabolism.

Compared to the genome-scale model solution space sampling results, where increased reaction fluxes were identified at higher carbon levels (except for three reactions, see Fig. 2), gene expression analysis identified specific reactions that were up- and downregulated. Additionally, RNA-Seq differential expression analysis identified reactions involved in glycan metabolism as being downregulated at high CO_2_ concentrations, whereas this group does not show up in the sampling results. Although N-glycan biosynthesis is included in the genome-scale model, glycans are not accounted for in the biomass reaction. Instead, a demand reaction was included in the model [DM_m2masn_c, demand for (GlcNAc)2(Man)3(Asn)1, KEGG glycan ID G10652] to account for the missing knowledge of the fraction of glycans present in the biomass. This demand reaction was blocked prior to the solution space sampling and thus cannot be accounted for in the sampling result.

**FIG 4:**
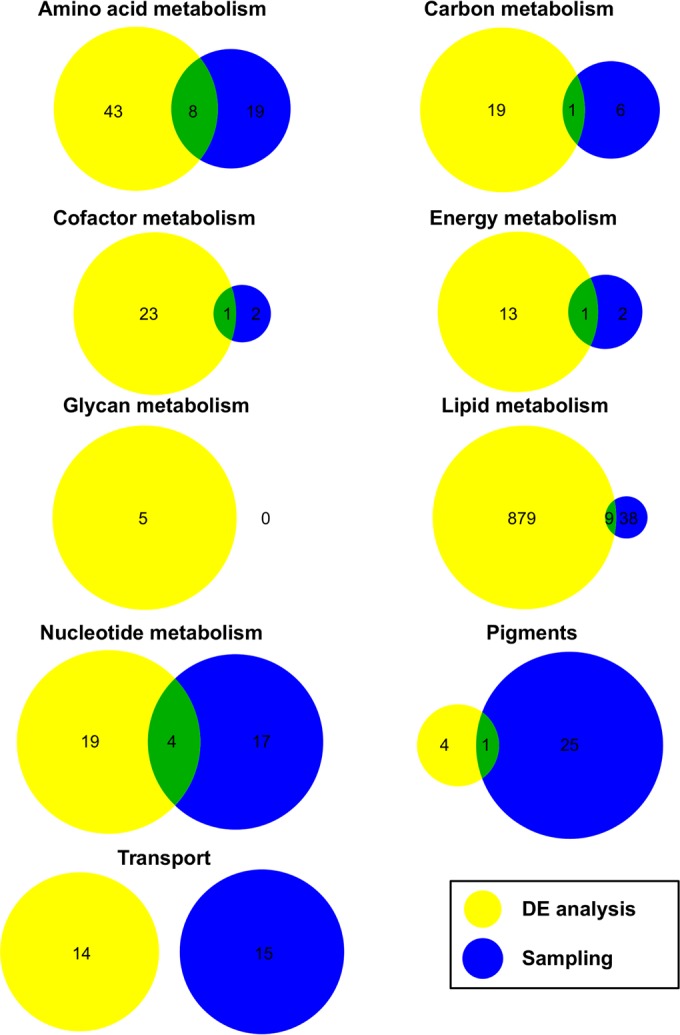
Comparison of reactions with different flux patterns. Reactions with different flux patterns (either up- or downregulated) at low and high carbon concentrations identified in the genome-scale metabolic model solution space sampling (blue) and the differential expression (DE, yellow) analysis are compared for each subsystem.

The sampling and differential expression analysis results also differ in the percentage of differentially expressed reactions per group. For example, about 10% of all reactions in pigment metabolism are differentially expressed according to RNA-Seq data, whereas the sampling results indicate that more than 40% of the reactions show different flux patterns. Although the same groups (except for glycan metabolism) are identified by the sampling and differential expression analysis, the reactions within these groups are different between the two analyses, as shown in Fig. 4.

The discrepancy in the metabolic response to rising CO_2_ levels, as observed between the metabolic model predictions and the differential expression analysis, might be explained by the different types of data exploited for the two analyses. The differential expression analysis is based on RNA-Seq data, whereas the metabolic model predictions are based on simulated reaction fluxes and thus protein activities. The differential expression analysis takes into account regulation by TFs but not posttranscriptional and -translational modifications, as well as allosteric regulation on the enzyme level. In contrast, the metabolic model does not include any regulatory effects and the predicted flux distributions are based on the mature enzyme activities, i.e., after modifications. However, random sampling of the metabolic model’s solution space identifies all possible flux distributions and, combined with further physiological data such as transcriptomics, more complete insights into the organism’s metabolism can be gained.

### Genome-scale model predicts effects of increasing carbon levels on nitrogen metabolism.

**FIG 5:**
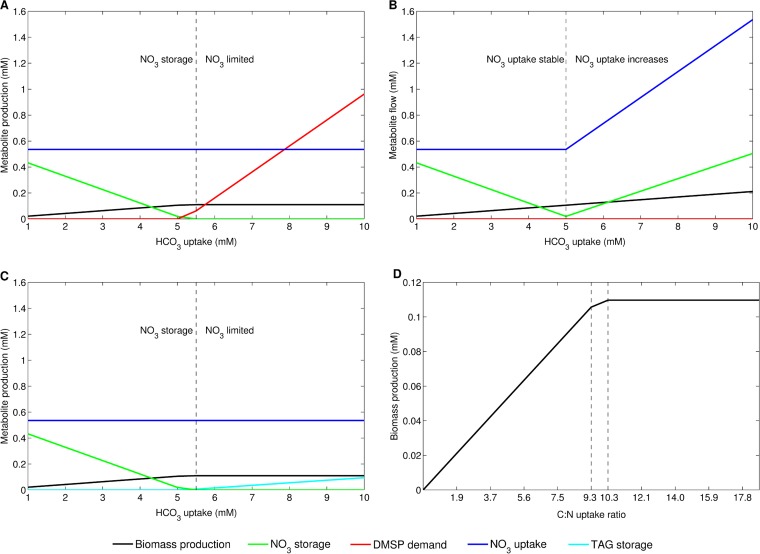
Model predictions at various HCO_3_ conditions. With increasing HCO_3_ concentrations, NO_3_ is limiting and is completely required for biomass production; subsequently, NO_3_ storage declines. Excess carbon is released as DMSP, which is the only reaction in the model allowing carbon secretion (A). Panel B shows biomass production and NO_3_ storage when NO_3_ uptake is increased linearly shortly before NO_3_ becomes limiting (at an HCO_3_ uptake level of 5 mM). Simulation results when carbon can be stored as chrysolaminarin or TAGs are depicted in panel C. Here, NO_3_ uptake is constrained and constant as in panel A. The model predicts that excess carbon is stored in the form of TAGs. (D) Effect of the carbon/nitrogen (C/N) ratio on biomass. Within this simulation, carbon uptake was varied from 0 to 10 mM and nitrogen uptake was kept constant at 0.535 mM. Biomass production increases at C/N ratios under 10.28 and stagnates at ratios over 10.28 because of limiting nitrogen availability. Note that for all of the simulations shown, the available CO_2_ was forced to be taken up.

The tight connection between carbon metabolism and nitrogen metabolism observed in the TRN is also represented in the genome-scale metabolic model of *P. tricornutum* (6). We investigated *P. tricornutum*’s behavior at different HCO_3_ levels simulating three different scenarios (Fig. 5). Scenario 1 implements a stepwise increase in the available HCO_3_ from 1 to 10 mM while nitrate uptake is constant. In scenario 2, HCO_3_ uptake increases stepwise and the nitrate supply is allowed to increase when the HCO_3_ level exceeds 5 mM. Scenario 3 includes demand reactions to mimic carbon and energy storage in the form of chrysolaminarin and TAG. The available HCO_3_ level increases stepwise, and nitrate uptake is kept constant.

According to the predictions of scenario 1 with stepwise increasing HCO_3_ uptake and a constant nitrate (NO_3_) supply, NO_3_ becomes limiting and is not stored internally with increasing HCO_3_ concentrations, as shown in Fig. 5A. Instead, under these conditions, the available NO_3_ is used for biomass production to enable maximal carbon fixation into biomass. Consequently, biomass production stagnates because of limiting NO_3_ availability. Since HCO_3_ further increases and the cell is forced to take it up, 3-(dimethylsulfonio)propanoate (DMSP) is secreted, which is the only reaction allowing the release of excess carbon.

Figure 5B demonstrates that the nitrogen supply, e.g., in the form of NO_3_, is indeed the limiting factor constraining biomass production using scenario 2 with stepwise increasing HCO_3_ uptake and allowing the NO_3_ supply to increase by 0.1 mM with each carbon uptake increase when the HCO_3_ uptake is ≥5.5 mM. The available NO_3_ is incorporated into biomass for HCO_3_ levels below 5 mM. The supplied rising nitrogen is sufficient to enable nitrogen storage and fixation of all presented carbon into biomass; thus, no DMSP is secreted.

In scenario 1, excess carbon was released in the form of DMSP. This reaction (DM_dmsp_c) was introduced into the model because of a knowledge gap in DMSP metabolism and to prevent its accumulation (6). In the third scenario, this demand reaction was replaced with reactions representing carbon and energy storage. Diatoms store excess carbon and energy in the form of chrysolaminarin, a β-1,3-glucose molecule representing the diatom storage glycan, and the storage lipid TAG. In scenario 3, carbon storage was enabled through the implementation of demand reactions for chrysolaminarin and TAG (16:1Δ9/16:1Δ9/16:0) (6). The simulation results are shown in Fig. 5C. Since NO_3_ uptake is constrained and constant throughout the simulation, nitrogen storage decreases with increasing HCO_3_ concentrations. At an HCO_3_ uptake of 5 mM, the biomass production increase is slowed down before it finally stagnates when the HCO_3_ uptake reaches 5.5 mM. NO_3_ storage shows a similar pattern and vanishes at HCO_3_ levels higher than 5.5 mM. With HCO_3_ uptake higher than 5.5 mM, excess carbon is stored as TAG, which increases with HCO_3_. In this scenario, excess carbon is not stored as chrysolaminarin.

Using the genome-scale metabolic network, we demonstrated that biomass production increases with elevating HCO_3_ levels until *P. tricornutum* encounters nitrogen limitation. A strong connection between carbon metabolism and nitrogen metabolism was also observed in the TRN, where we showed that genes involved in nitrogen and carbon metabolism are often coregulated. It should be noted that temperature, nutrient deprivation, and other factors are also known to influence the growth of *P. tricornutum* but were not taken into account in this study. Our work describes for the first time which genes are coexpressed and coregulated upon different environmental perturbations and provides insight into the effect the limitation of a nutrient such as carbon or nitrogen has on the phenotype. The integration of the regulatory network developed here and the published metabolic model offers new insights into the model diatom *P. tricornutum*’s metabolic and regulatory interactions and provides a comprehensive framework of responses to increasing atmospheric carbon levels.

## MATERIALS AND METHODS

### RNA-Seq preparation and differential expression analysis.

Raw read counts were read into R version 3.2.3 (https://www.r-project.org), scaled by using DESeq scaling factors (16), log_2_ transformed (a constant +1 was added prior to log_2_ transformation), and adjusted for batch effects introduced by the utilization of different sampling platforms with the ComBat software (17). An overview of the transcriptome-wide RNA-Seq expression data exploited for the diverse environmental responses used in this study is shown in Table 1.

To filter the transcriptomic data set for metabolic genes, the genome-scale model of *P. tricornutum* was used (6). Known TFs were translated into Phatr3 gene IDs (http://protists.ensembl.org/Phaeodactylum_tricornutum/Info/Index) by using an in-house mapping table. The expression profiles of 196 known TFs in *P. tricornutum* (18) were added to the filtered data set, which comprised 1,214 genes and 179 experimental samples in total.

Differential expression analysis at normal (400 ppm) and high (5,000 ppm) CO_2_ levels was performed with the R/Bioconductor package limma (19). The Benjamini-Hochberg method was chosen to adjust the *P* value to a false-discovery rate of 5%.

### Detection of coregulated gene modules and their regulatory influences.

The cMonkey_2_ algorithm discovers coregulated modules in transcriptome profiles by integrating additional information, such as the genome sequence, protein-protein interactions, and the *de novo* detection of *cis*-regulatory DNA sequence motifs, and aggregates it into a combined score to improve clustering (13, 14).

Since *P. tricornutum* is not part of the RSAT database from which cMonkey_2_ retrieves all of its organism information, the input files were assembled before running cMonkey_2_. The genome annotation of *P. tricornutum* version 3 (Phatr3) was obtained from Ensembl Protist (http://protists.ensembl.org/Phaeodactylum_tricornutum/Info/Index). Chloroplastic and mitochondrial genomic information was obtained from NCBI (GenBank accession no. NC_008588 and HQ840789, respectively) (20, 21). The functional annotation was gathered from the genome-scale model of *P. tricornutum* (6). Protein-protein interactions were obtained from the STRING database version 10 (22) and replaced with the corresponding Phatr3 gene IDs with an in-house Phatr2-to-Phatr3 gene ID mapping table. The cMonkey_2_ algorithm was run for 2,000 iterations and generated 121 biclusters with a mean of 20 genes per cluster.

The Inferelator algorithm was used to infer the regulatory influences of 196 TFs influencing the expression of the coregulated modules discovered by cMonkey_2_ (9).

### GO enrichment analysis.

The Python tool goatools version 0.5.9 was used to find over- and underrepresented GO terms in the 121 coregulated modules generated on the basis of Fisher’s exact test (23). On the basis of the gene-to-subsystem assignment in the metabolic network of *P. tricornutum* (6), each gene was assigned a GO term. The option to propagate counts to all of the parents of a GO term was disabled. To correct for multiple-hypothesis testing, a false-discovery rate correction was applied. We found significant over- and underrepresentation of 59 GO terms in 112 out of the 121 biclusters.

### Connectivity analysis.

On the basis of the clustering result and the *P. tricornutum* genome-scale model (6), a cluster-gene reaction product association list was compiled. On the basis of this list, for each cluster, the substrates and products were extracted. In the case of reversible reactions, the substrates can also be products and vice versa. All currency metabolites, i.e., water, ATP, ADP, AMP, NAD(P)^+^, NAD(P)H, protons, oxygen, inorganic phosphate, pyrophosphate, and CO_2_, were removed from the cluster-substrate-product list.

Highly connected clusters were identified on the basis of substrates or products. The connectivity of two clusters was defined as the number of common substrates (or products) divided by the total number of substrates (or products) in both clusters. Here, two clusters are strongly connected if their connectivity is >0.9.

### Modeling simulations.

Modeling simulations were performed with the Gurobi Optimizer version 5.6.3 (Gurobi Optimization Inc., Houston, TX) solver in MatLab (the MathWorks Inc., Natick, MA) with the COBRA Toolbox (24). Nutrient uptake (Table 2) and the BOF were set according to experimental data as described in reference 6.

**TABLE 2:** Constraints applied to the metabolic network of *P. tricornutum*^a^

Reaction ID	Applied constraint (mM)
Ex_hco3_e	LB^b^ and UB,^c^ −1.57 for low HCO_3_, −3.14 for high HCO_3_
Ex_no3_e	LB and UB, −0.535
Ex_biotin_e	LB, −1,000; UB, 0
Ex_fe2_e	LB, −1,000; UB, 0
Ex_h_e	LB, −1,000; UB, 1,000
Ex_h2o_e	LB, −1,000; UB, 1,000
Ex_o2_e	LB, −1,000; UB, 1,000
Ex_pi_e	LB, −0.22; UB, 0
Ex_na1_e	LB, −1,000; UB, 1,000
Ex_so4_e	LB, −28.8; UB, 0
Ex_mg2_e	LB, −1,000; UB, 0
Ex_cl_e	LB, −1,000; UB, 1,000
Ex_thm_e	LB, −1,000; UB, 0

a Constraints were applied to nutrient uptake and product secretion for sampling and simulations as used in reference 6, except for HCO_3_ instead of CO_2_ uptake. Exchange reactions not shown here are blocked.

b LB, lower bound.

c UB, upper bound.

### Sampling of solution space.

To uniformly sample the solution space of the *P. tricornutum* metabolic network iLB1027_lipid (6), optGpSampler (25) for MatLab (the MathWorks Inc., Natick, MA) with Gurobi Optimizer version 6.5.0 (Gurobi Optimization Inc., Houston, TX) was used. Two different carbon conditions were sampled, low and high. HCO_3_ uptake was set to 1.57 mM and doubled to 3.14 mM for the simulations of high-carbon conditions. All other constraints were identical between the two sampled conditions (Table 2). Before sampling, all of the reactions and metabolites that could not carry flux under the simulated conditions were removed from the models. To constrain the genome-scale model more by knocking out reactions associated with nonexpressed genes, our expression data were used to obtain genes not expressed under normal (400 ppm)- and high (5,000 ppm)-CO_2_ conditions. Corresponding reactions and their gene-reaction associations were extracted from the genome-scale model. However, this analysis did not result in any reaction knockout. The reduced metabolic model contains 3,904 reactions and 1,734 metabolites. Both networks, representing low- and high-carbon conditions, were sampled by using 50,000 sample points with a step count of 7,808, which is double the number of reactions in the model.

To determine if the flux distributions at low and high HCO_3_ levels are significantly different, the minimal distance between the histograms was determined for each reaction in the metabolic model by randomly permuting the flux vectors and subtracting them from each other. To get a representative distance, this procedure was repeated 100 times, yielding 100 distance vectors for each histogram comparison. The mean of the minimum number of positive and negative entries in each distance vector was used to calculate the *P* value for the two-sided test. To correct for multiple-hypothesis testing, a false-discovery rate correction was applied, setting the significance threshold at 0.05.

## References

[1] Caldeira K, Wickett ME 2003 Oceanography: anthropogenic carbon and ocean pH. Nature 425:365. doi:10.1038/425365a. 14508477

[2] Bowler C, Vardi A, Allen AE 2010 Oceanographic and biogeochemical insights from diatom genomes. Annu Rev Mar Sci 2:333–365. doi:10.1146/annurev-marine-120308-081051. 21141668

[3] Falkowski PG, Raven JA 1997 Aquatic photosynthesis. Blackwell Scientific Publications, Hoboken, NJ.

[4] Nelson DM, Tréguer P, Brzezinski MA, Leynaert A, Quéguiner B 1995 Production and dissolution of biogenic silica in the ocean: revised global estimates, comparison with regional data and relationship to biogenic sedimentation. Global Biogeochem Cycles 9:359–372. doi:10.1029/95GB01070.

[5] Hopkinson BM, Dupont CL, Matsuda Y 2016 The physiology and genetics of CO_2_ concentrating mechanisms in model diatoms. Curr Opin Plant Biol 31:51–57. doi:10.1016/j.pbi.2016.03.013. 27055267

[6] Levering J, Broddrick J, Dupont CL, Peers G, Beeri K, Mayers J, Gallina AA, Allen AE, Palsson BO, Zengler K 2016 Genome-scale model reveals metabolic basis of biomass partitioning in a model diatom. PLoS One 11:e0155038. doi:10.1371/journal.pone.0155038. 27152931 PMC4859558

[7] Kim TY, Sohn SB, Kim YB, Kim WJ, Lee SY 2012 Recent advances in reconstruction and applications of genome-scale metabolic models. Curr Opin Biotechnol 23:617–623. doi:10.1016/j.copbio.2011.10.007. 22054827

[8] Lewis NE, Nagarajan H, Palsson BO 2012 Constraining the metabolic genotype-phenotype relationship using a phylogeny of in silico methods. Nat Rev Microbiol 10:291–305. doi:10.1038/nrmicro2737. 22367118 PMC3536058

[9] Bonneau R, Reiss DJ, Shannon P, Facciotti M, Hood L, Baliga NS, Thorsson V 2006 The Inferelator: an algorithm for learning parsimonious regulatory networks from systems-biology data sets de novo. Genome Biol 7:R36. doi:10.1186/gb-2006-7-5-r36. 16686963 PMC1779511

[10] Weyman PD, Beeri K, Lefebvre SC, Rivera J, McCarthy JK, Heuberger AL, Peers G, Allen AE, Dupont CL 2015 Inactivation of Phaeodactylum tricornutum urease gene using transcription activator-like effector nuclease-based targeted mutagenesis. Plant Biotechnol J 13:460–470. doi:10.1111/pbi.12254. 25302562

[11] Smith SR, Abbriano RM, Hildebrand M 2012 Comparative analysis of diatom genomes reveals substantial differences in the organization of carbon partitioning pathways. Algal Res 1:2–16. doi:10.1016/j.algal.2012.04.003.

[12] Bailleul B, Berne N, Murik O, Petroutsos D, Prihoda J, Tanaka A, Villanova V, Bligny R, Flori S, Falconet D, Krieger-liszkay A, Santabarbara S, Rappaport F, Joliot P, Tirichine L, Falkowski PG, Cardol P, Bowler C, Finazzi G 2015 Energetic coupling between plastids and mitochondria drives CO_2_ assimilation in diatoms. Nature 524:366–369. doi:10.1038/nature14599. 26168400

[13] Reiss DJ, Baliga NS, Bonneau R 2006 Integrated biclustering of heterogeneous genome-wide datasets for the inference of global regulatory networks. BMC Bioinformatics 7:280. doi:10.1186/1471-2105-7-280. 16749936 PMC1502140

[14] Reiss DJ, Plaisier CL, Wu WJ, Baliga NS 2015 cMonkey2: automated, systematic, integrated detection of co-regulated gene modules for any organism. Nucleic Acids Res 43:e87. doi:10.1093/nar/gkv300. 25873626 PMC4513845

[15] Orth JD, Thiele I, Palsson BØ 2010 What is flux balance analysis? Nat Biotechnol 28:245–248. doi:10.1038/nbt.1614. 20212490 PMC3108565

[16] Anders S, Huber W 2010 Differential expression analysis for sequence count data. Genome Biol 11:R106. doi:10.1186/gb-2010-11-10-r106. 20979621 PMC3218662

[17] Johnson WE, Li C, Rabinovic A 2007 Adjusting batch effects in microarray expression data using empirical Bayes methods. Biostatistics 8:118–127. doi:10.1093/biostatistics/kxj037. 16632515

[18] Rayko E, Maumus F, Maheswari U, Jabbari K, Bowler C 2010 Transcription factor families inferred from genome sequences of photosynthetic stramenopiles. New Phytol 188:52–66. doi:10.1111/j.1469-8137.2010.03371.x. 20646219

[19] Ritchie ME, Phipson B, Wu D, Hu Y, Law CW, Shi W, Smyth GK 2015 Limma powers differential expression analyses for RNA-sequencing and microarray studies. Nucleic Acids Res 43:e47. doi:10.1093/nar/gkv007. 25605792 PMC4402510

[20] Oudot-Le Secq MP, Grimwood J, Shapiro H, Armbrust EV, Bowler C, Green BR 2007 Chloroplast genomes of the diatoms Phaeodactylum tricornutum and Thalassiosira pseudonana: comparison with other plastid genomes of the red lineage. Mol Genet Genomics 277:427–439. doi:10.1007/s00438-006-0199-4. 17252281

[21] Oudot-Le Secq MP, Green BR 2011 Complex repeat structures and novel features in the mitochondrial genomes of the diatoms Phaeodactylum tricornutum and Thalassiosira pseudonana. Gene 476:20–26. doi:10.1016/j.gene.2011.02.001. 21320580

[22] Szklarczyk D, Franceschini A, Wyder S, Forslund K, Heller D, Huerta-Cepas J, Simonovic M, Roth A, Santos A, Tsafou KP, Kuhn M, Bork P, Jensen LJ, von Mering C 2015 STRING v10: protein-protein interaction networks, integrated over the tree of life. Nucleic Acids Res 43:D447–D452. doi:10.1093/nar/gku1003. 25352553 PMC4383874

[23] Tang H, Klopfenstein D, Pedersen B, Flick P, Sato K, Ramirez F, Yunes J, Mungall C 30 9 2015 GOATOOLS: tools for gene ontology. Zenodo, European Organization for Nuclear Research, Geneva, Switzerland https://zenodo.org/record/31628#.WIpIqk3JCos.

[24] Schellenberger J, Que R, Fleming RMT, Thiele I, Orth JD, Feist AM, Zielinski DC, Bordbar A, Lewis NE, Rahmanian S, Kang J, Hyduke DR, Palsson BØ 2011 Quantitative prediction of cellular metabolism with constraint-based models: the COBRA toolbox v2.0. Nat Protoc 6:1290–1307. doi:10.1038/nprot.2011.308. 21886097 PMC3319681

[25] Megchelenbrink W, Huynen M, Marchiori E 2014 optGpSampler: an improved tool for uniformly sampling the solution-space of genome-scale metabolic networks. PLoS One 9:e86587. doi:10.1371/journal.pone.0086587. 24551039 PMC3925089

