# Supplementary figures and images for: Integrated Regulatory and Metabolic Networks of the Marine Diatom Phaeodactylum tricornutum Predict the Response to Rising CO2 Levels
Source: mSystems. 2017 Feb 14;2(1):e00142-16. doi: 10.1128/mSystems.00142-16 (PMC5309336; doi:10.1128/mSystems.00142-16)

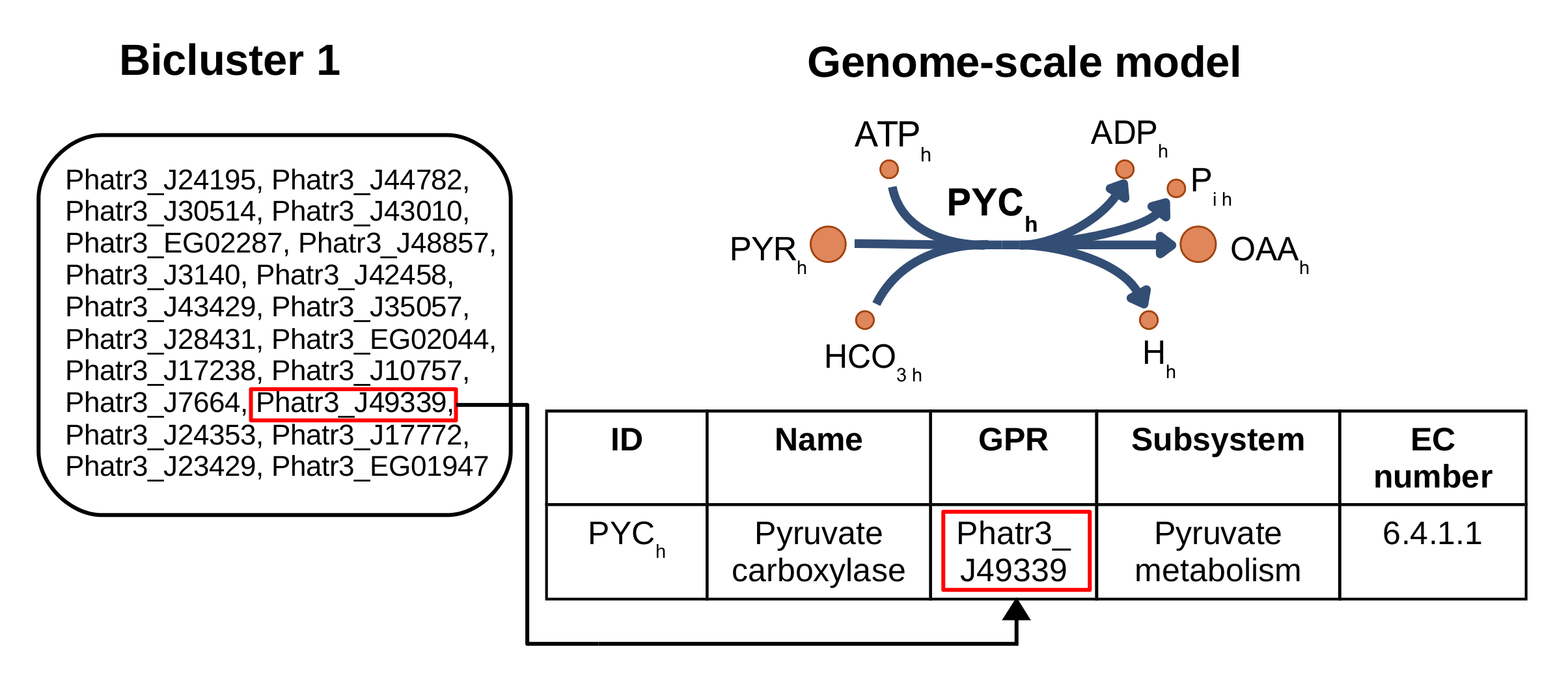

Supplement: FIG S1 [file sys001172087sf6.tif]
